# Comment on “Second-Hand Smoking among Intermediate and Secondary School Students in Madinah, Saudi Arabia”

**DOI:** 10.1155/2015/952450

**Published:** 2015-10-26

**Authors:** Siddharudha Shivalli

**Affiliations:** Department of Community Medicine, Yenepoya Medical College, Yenepoya University, Mangalore, Karnataka 575018, India

I read the article titled “Second-Hand Smoking among Intermediate and Secondary School Students in Madinah, Saudi Arabia” by Al-Zalabani et al. [1], with curiosity. Authors' efforts are admirable. This study provides valuable information for the evidence based fine tuning of present smoking legislation in the study area. However, the following issues and concerns need to be addressed.

Knowledge about harmful effects of smoking from family or school (yes versus no) and belief in the negative effects of second-hand smoking (yes versus no) have been considered as independent variables in this study. What questions were asked and which criteria were used to classify the study participant as “knowledgeable about the harmful effects smoking” and “believer in negative effects of second-hand smoking”? This should have been explicitly mentioned to avoid ambiguity.

The estimated prevalence of second-hand smoking exposure among the studied adolescents at home, outside households, and overall exposure should have been reported with 95% confidence intervals [2]. In Table 1, authors have repeatedly mentioned *P* value as “0.00”. SPSS, by default setting, displays *P* value as zero if it extends beyond 3 decimal points (i.e., *P* = 0.0000002 would be displayed as *P* = 0.00). Practically, the value of *P* cannot be zero and hence, I would suggest to report it as *P* < 0.0001.

Authors have included all the independent variables in regression model and associations are expressed as adjusted OR with 95% confidence intervals. However, adequacy of the applied binary logistic regression model is not mentioned. Failure to do so may lead to misleading or incorrect inferences. Although the study sample was large, a word about *R* ^2^ (explaining the variance in the outcome variable) of the applied regression model would have been more affirmative.

In the discussion, authors should have given logical explanations about how the significant correlates in regression analysis are associated with second-hand smoking exposure. For example, why boys (versus girls) and those who do not stay with parents are more likely to be exposed to second-hand smoking? Intuitive epidemiological reasoning of the aforesaid, keeping the sociocultural background in mind, would have been more informative and interesting.

In conclusion, authors mention that, in Moslem communities, mosques appeared to have a crucial role in raising the awareness of youth and their families not only on the harmful effect of smoking, but also on religion's stance on this habit. Such recommendation is to be endorsed; however, it must be supported by proper reference/s. An additional limitation stating that not all the observed associations infer causality due to cross-sectional study design should have been mentioned.

Nonetheless, I must applaud the authors for investigating an important public health problem on a large scale.
